# The influence of vitamin D analogs on calcification modulators, N-terminal pro-B-type natriuretic peptide and inflammatory markers in hemodialysis patients: a randomized crossover study

**DOI:** 10.1186/1471-2369-15-130

**Published:** 2014-08-12

**Authors:** Ditte Hansen, Knud Rasmussen, Lars M Rasmussen, Helle Bruunsgaard, Lisbet Brandi

**Affiliations:** 1 Department of Nephrology, Rigshospitalet, Blegdamsvej 9, 2100 Copenhagen, Denmark; 2 Department of Medicine, Roskilde Hospital, Koegevej 7-13, 4000 Roskilde, Denmark; 3 Department of Clinical Biochemistry and Pharmacology, Odense University Hospital, Sdr. Boulevard 29, DK-5000 Odense, Denmark; 4 Center of Inflammation and Metabolism, Rigshospitalet, Blegdamsvej 9, 2100 Copenhagen, Denmark; 5 Department of Medicine, Hillerød Hospital, Dyrehavevej 29, DK-3400 Hillerød, Denmark

**Keywords:** Cardiovascular disease, Heart failure, Hemodialysis, Vascular calcification, Vitamin D

## Abstract

**Background:**

The risk of cardiovascular disease is tremendously high in dialysis patients. Dialysis patients treated with vitamin D analogs show decreased cardiovascular morbidity and mortality compared with untreated patients. We examined the influence of two common vitamin D analogs, alfacalcidol and paricalcitol, on important cardiovascular biomarkers in hemodialysis patients. Anti-inflammatory effects and the influence on regulators of vascular calcification as well as markers of heart failure were examined.

**Methods:**

In 57 chronic hemodialysis patients enrolled in a randomized crossover trial comparing paricalcitol and alfacalcidol, we examined the changes in osteoprotegerin, fetuin-A, NT-proBNP, hs-Crp, IL-6 and TNF-α, during 16 weeks of treatment.

**Results:**

NT-proBNP and osteoprotegerin increased comparably in the paricalcitol and alfacalcidol-treated groups. Fetuin-A increased significantly in the alfacalcidol-treated group compared with the paricalcitol-treated group (difference 32.84 μmol/l (95% C.I.; range 0.21–67.47)) during the first treatment period. No difference was found between the groups during the second treatment period, and IL-6, TNF-α and hs-Crp were unchanged in both treatment groups.

**Conclusions:**

Paricalcitol and alfacalcidol modulate regulators of vascular calcification. Alfacalcidol may increase the level of the calcification inhibitor fetuin-A. We did not find any anti-inflammatory effect or difference in changes of NT-proBNP.

**Trial registry:**

ClinicalTrials.gov NCT00469599 May 3 2007.

## Background

Cardiovascular mortality in hemodialysis patients is tremendously high compared with the general population [1]. Vitamin D deficiency is associated with increased risk of cardiovascular disease and mortality in hemodialysis patients [2-5]. Observational studies have shown that hemodialysis patients treated with vitamin D analogs have an increased survival [6,7]. It has been proposed that the survival advantage differs between various vitamin D analogs [8].

The mechanism of cardiovascular protection by vitamin D analogs has been proposed to be due to anti-inflammatory properties [9], modulation of calcification factors and interaction in the bone-vascular crosstalk [10,11], and modulation of cardiac structure and function [12-14].

In order to explore the influence of vitamin D analogs in hemodialysis patients, we examined the changes in inflammation (hs-Crp, IL-6 and TNF-α), cardiac function (Nt-proBNP) and calcification factors (fetuin-A and osteoprotegerin) during treatment with the vitamin D analogs alfacalcidol and paricalcitol, and the possible differences between the effects of these analogs.

## Methods

### Subjects

Subjects in this study were a subset of participants (57 of 86 enrolled patients), in a multicenter, open-label, 1:1 randomized, crossover study comparing alfacalcidol and paricalcitol (SHPT-study). These subjects and the primary results of the study have been described elsewhere [15,16]; in brief, adults receiving chronic hemodialysis therapy with well-controlled calcium and phosphate levels, and secondary hyperparathyroidism (p-iPTH >350 pg/ml) received alfacalcidol or paricalcitol for 16 weeks in order to control the secondary hyperparathyroidism.

Only participants from whom blood had been collected during the entire trial period were included. The entire study population did not have blood sample collection because of: 1) improper collection at study sites; 2) participants refusing to participate in Biobank collection; and 3) withdrawal from the original study.

The study was in compliance with the Helsinki Declaration of 1975, revised in 2000, and was approved by the Danish National Committee on Biomedical Research Ethics (SJ-27). All participants had received written and oral information prior to the study and had given written informed consent.

### Design

The design has been described previously and discussed in detail [15]. The study took place in 10 public Danish dialysis departments, all being a part of hospital nephrology departments. The randomization was performed after a 6-week washout period. The first arm received alfacalcidol for 16 weeks and then went through another 6-week washout period before they received paricalcitol for 16 weeks. The second arm received paricalcitol for 16 weeks followed by a 6-week washout period before they received alfacalcidol for 16 weeks. The starting dose of alfacalcidol (Etalpha®Injection) was 3 μg per week and the starting dose of paricalcitol (Zemplar®Injection) was 9 μg per week. Every second week the dose was titrated 50% according to p-phosphate, p-calcium and p-iPTH. As long as p-phosphate was <1.8 mmol/l, ionized p-calcium was <1.30 mmol/l and p-iPTH was >150 pg/ml, the dose was increased. When p-iPTH was ≤150 pg/ml, p-phosphate was <1.8 mmol/l and ionized p-calcium was <1.35 mmol/l, the dose was maintained. If at any time p-phosphate was >1.8 mmol/l or ionized p-calcium was >1.35 mmol/l in two repeated measurements, the dose was reduced. The minimal dose of alfacalcidol was 1.5 μg/week and that of paricalcitol was 4.5 μg/week. If further reduction was needed, treatment was paused.

After inclusion, the dose of calcium-containing phosphate-binders could only be reduced or left unchanged. Elevated p-phosphate levels were treated thoroughly with calcium-free phosphate-binders, dietary intervention and re-evaluation of the dialysis dose. Elevated p-calcium would lead to dietary intervention and reduction of calcium containing phosphate binders. The calcium concentration of the dialysate was fixed at 1.25 mmol/l.

Blood samples were collected at the beginning (weeks 6 and 22) and at the end (weeks 28 and 44) of each treatment period, separated by a 6-week washout period. Blood samples were drawn from blood lines of the dialyzer before the start of dialysis. Samples were frozen and collected at Roskilde County Hospital. At the end of the study, samples were aliquoted and cooled, and sent to Odense University Hospital or Rigshospitalet for analysis.

### Osteoprotegerin (OPG)

Plasma OPG was measured using a sandwich enzyme-linked immunoabsorbent assay (ELISA) with commercially available antibodies (R&D Systems, Minneapolis, MN) and modified antibodies as previously described [17,18]. In summary, mouse anti-human OPG was used as the capture antibody, and a biotinylated goat anti-human OPG, in combination with an Eu-labeled streptavidin, was used for detection. Recombinant human OPG was used for calibration and the analytical range of the assay was 62.5–4,000 pg/ml. Washing steps were performed on an automated wash system (1296-026 Delfia Platewasher, Wallac, Waltham, MA, USA) and optical densities were determined at 450 nm on a VICTOR X5 Multimode Plate Reader (Perkin-Elmer, Waltham, MA, USA). Data were plotted with a four-parameter logistic (4PL) curve fit of OD readings using GraphPad Prism software (version 5; GraphPad Software, La Jolla, CA). Our intra-assay coefficient of variation was 3%, and the inter-assay variation was 8% in duplicate measurements.

### Fetuin-A

A commercially available ELISA kit for human fetuin-A with monoclonal antibodies (R&D Quantikine, catalog no. DFTA00) was used. NS0-expressed recombinant human fetuin-A was used for calibration and plasma was measured in a 4,000-fold dilution to read within the linear range of the standard curve (75–1,000 pg/ml). Washing steps were performed on an automated wash system (1296-026 Delfia Platewasher) and optical densities were determined at 450 nm on a VICTOR X5 Multimode Plate Reader (Perkin-Elmer). Data were plotted with a four-parameter logistic (4PL) curve fit of OD readings using GraphPad Prism software (version 5; GraphPad Software). Our intra-assay coefficient of variation was 5%, and the inter-assay variation was 9% in duplicate measurements.

### N-terminal pro-brain natriuretic peptide (NT-proBNP)

Measurement of NT-proBNP was done with a Cobas e411 instrument (Roche). Imprecision of the analysis was below 10%.

### High-sensitivity C-reactive protein (Hs-Crp)

Hs-Crp was measured on an Architect C8000 (Abbott Laboratories, Chicago, USA) using the latex immunoassay Crp *Vario* (Sentinel Diagnostics, Milan, Italy). Reaction parameters were applied to the Architect C8000 system as recommended by the supplier. Within-run/between-run coefficients of variations for the Crp *Vario* assay on the C8000 were <2.3% and <4.3%, respectively.

### Interleukin-6 (IL-6) and tumor necrosis factor-α (TNF-α)

An electro-chemiluminescence multiplex system was used on a Sector 2400 Imager from Meso Scale Discovery (Gaithersburg, MD, USA) according to the manufacturer’s instructions. IL-6 and TNF-α were measured using a multiplex system. All samples were run as duplicates.

### Fibroblast growth factor (FGF-23)

FGF23 was measured using a sandwich enzyme-linked immunosorbent assay (Kainos Laboratories Inc., Tokyo, Japan), which detects only the biologically active intact FGF23. The intra- and inter-assay coefficients of variation were less than 5.0%.

### Other laboratory parameters

P-iPTH, ionized p-calcium and p-phosphate were measured every second week during treatment periods in order to guide dose adjustments. These were then analyzed at participating department local laboratories.

### Statistical analysis

The distribution of variables and changes in the variables from the start until the end was described. Continuous data were described as mean (standard deviation or SEM) if normally distributed or median (range) if not. Categorical data were described as numbers and percentages. Comparison of changes between groups were performed according to Altman *et al.*[19]. Parametric tests were used if the distributions were normal (paired and unpaired t-tests) and non-parametric tests (Fischer’s Exact test, Mann-Whitney U and Wilcoxon signed-rank test) were used for ordered or continuous data. NT-proBNP and hs-Crp were logarithmic transformed in order to reach normality. Explanatory factors for changes in OPG, fetuin-A and NT-proBNP were found using multivariate analysis of repeated measurements with backward selection. In order to explore whether the presence of infection influenced the inflammatory markers, patients experiencing at least one adverse event of infection during the study were censored, and the data were analyzed in remaining patients. All tests were two-sided tests (α = 0.05). Analysis was performed using the SAS 9.1 software package (SAS Institute Inc., Cary, NC, USA).

## Results

### Patient characteristics

**Figure 1:**
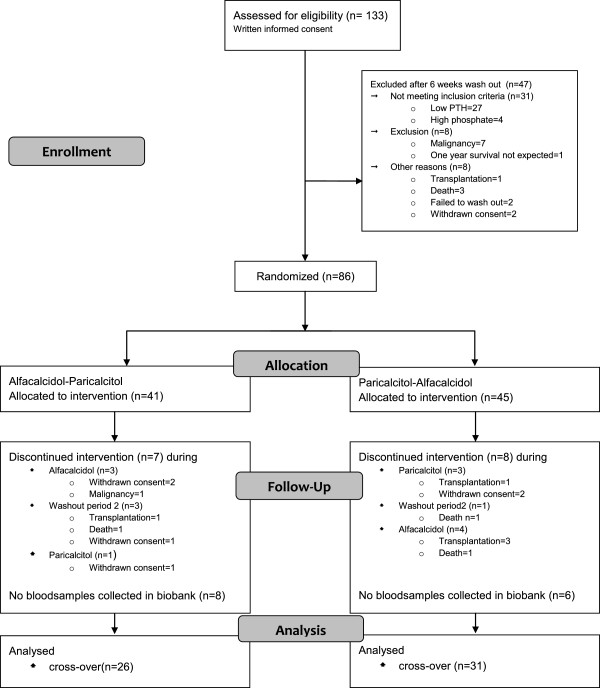
Patients flow diagram.

In 57 patients, blood samples from all four visits during the randomized controlled SHPT study were available (Figure 1). There was no difference between patient characteristics of the present study and enrolled patients in the SHPT study [16]. There was no difference in patient characteristics between treatment groups (Table 1).

**Table 1:** Baseline characteristics

	**Alfacalcidol-paricalcitol**	**Paricalcitol-alfacalcidol**
	**n = 26**	**n = 31**
Age (years ± SD)	66.7 ± 10.8	67.1 ± 13.1
Gender (male/female)	17/9	17/12
Race: Caucasian	26(100%)	29(100%)
Time on dialysis (month; median (range))	36.5 (4-236)	36.0(3-132)
Etiology of ESRD		
Diabetes	1(4%)	5(17%)
Nephrosclerosis	4(15%)	6(21%)
Polycystic	5(19%)	3(10%)
Chronic glomerulonephritis	5(19%)	4(14%)
Chronic interstitial	2(8%)	2(7%)
Postrenal	4(15%)	3(10%)
Unknown	5(19%)	6(21%)
Cardiovascular history		
Hypertension	20(77%)	22(76%)
Diabetes	2(8%)	5(17%)
Ischemic heart disease	9(35%)	10(34%)
Stroke or TCI	3(12%)	7(24%)
Heart failure	4(15%)	4(14%)
Biochemical		
s-FGF23 (pg/ml (median (range)))	2952(95-25543)	2480(117-32650)
p-intact PTH (pg/ml ± SD)	568(246)	523(175)
p-calcium ion (mmol/l ± SD)	1.16(0.08)	1.15(0.08)
p-phosphate (mmol/l ± SD)	1.50(0.23)	1.49(0.27)
p-25hydroxyvitamin D2 + D3 (nmol/l ± SD)	43.4(24.3)	34.3(22.0)

### Changes in mineral metabolism

Changes in. iPTH, ionized calcium and phosphate (FGF23 has been reported previously [16,20]).

Changes in NT-proBNP, OPG, fetuin-A, IL-6, TNF-α and hs-Crp are shown in Table 2.

**Table 2:** NT-proBNP, inflammatory markers, osteoprotegerin and fetuin-A during treatment with vitamin D analogs

	**Period 1**							**Period 2**						
	**Alfacalcidol**			**Paricalcitol**			**Difference between groups**	**Alfacalcidol**			**Paricalcitol**			**Difference between groups**
	**Week 6**	**Week 22**	**Increase**	**Week 6**	**Week 22**	**Increase**		**Week 28**	**Week 44**	**Increase**	**Week 28**	**Week 44**	**Increase**	
NT-proBNP (pmol/l) median (range)	417.8 (78-19048) N = 26	606.2 (90-63974)	187.7 (-888-44932)	485.4 (79-13543) N = 29	785.5 (39-7766)	300.1 (-12433-6124)*	-121.1 (95%CI -429.4 to 187.2)	763.8 (55-2769) N = 29	1005.0 (43-8519)	155.8 (-1917-7680)	497.1 (54-42379) N = 26	487.6 (45-53669)	-42.1 (-3262-11290)	-429.7 (95%CI -938.1 to 78.8)
Log NT-proBNP	2.74 (0.60) N = 26	6.63 (1.47)	**3.89 (0.19)#**	2.76 (0.57) N = 29	6.69 (1.22)	**3.93 (0.17)#**	0.04 (95%CI -0.54 to 0.47)	6.39 (1.07) N = 29	6.73 (1.21)	0.34 (0.19)	6.60 (1.53) N = 26	6.37 (1.49)	-0.22 (0.13)	**-0.56 (95**% **CI -1.04 to -0.10)¤**
hs-Crp (mg/l) median (range)	9.61 (0.3 -28.0) N = 24	6.58 (0.4-73.2)	-0.07 (-21-63)	7.23 (0.3-27.7) N = 29	5.30 (0.3-81.0)	-0.01 (-21-66)	-0.76 (95%CI -5.9 to 4.4)	7.52 (0.6-62.4) N = 29	5.25 (0.2-34.3)	-0.31 (-55-11)	6.96 (0.25-48.3) N = 24	5.46 (0.37-60.8)	-1.18 (-46- 58)	-0.85 (95%CI -4.7 to 3.0)
Log hs-Crp	1.86 (1.20) N = 24	1.80 (1.38)	-0.06 (0.21)	1.54 (1.31) N = 29	1.58 (1.54)	0.03 (0.21)	0.09 (95%CI -0.69 to 0.51)	1.70 (1.29) N = 29	1.38 (1.38)	-0.32 (0.19)	1.93 (1.26) N = 24	1.67 (1.08)	-0.27 (0.27)	0.05 (95%CI -0.60 to 0.71)
IL-6 (pg/ml)	6.99 (5.34) N = 21	7.05 (8.40)	0.06 (1.90)	5.56 (3.68) N = 24	8.34 (14.24)	2.79 (2.64)	-2.73 (95%CI -9.47 to 4.01)	6.59 (3.51) N = 21	5.66 (5.21)	-0.93 (0.75)	6.11 (3.91) N = 24	5.90 (3.53)	-0.21 (1.07)	0.72 (95%CI -1.87 to 3.29)
TNF-α (pg/ml)	6.63 (2.45) N = 23	7.76 (6.16)	1.13 (1.32)	7.05 (2.20) N = 26	6.85 (2.28)	-0.21 (0.44)	1.34 (95%CI -1.33 to 4.00)	6.59 (2.36) N = 26	7.18 (3.00)	0.53 (1.32)	6.42 (2.26) N = 23	7.04 (2.19)	-1.22 (2.16)	- 1.75 (95%CI -6.72 to 3.21)
Fetuin-A (μg/ml)	319.15 (76.41) N = 23	362.92 (77.77)	**43.77 (14.07)#**	330.81 (81.83) N = 26	340.75 (84.98)	9.93 (9.62)	**32.84 (95%CI 0.21 to 67.47) ¤**	331.29 (107.15) N = 26	318.94 (91.32)	-12.35 (18.39)	329.55 (81.00) N = 23	299.20 (74.95)	-30.35 (22.26)	-42.7 (95%CI -75.62 to 33.69)
Osteoprotegerin (pg/ml)	4461.77 (1905.1), N = 22	4878.00 (1954.20)	**416.23 (178.52)#**	4009.39 (1636.80) N = 26	4442.15 (2147.7)	**432.77 (193.65)#**	16.54 (95%CI -544.13 to 521.05)	4333.4 (1947.1) N = 26	4567.04 (2003.1)	**233.64 (102.55)#**	4982.68 (1988.9) N = 22	4911.09 (1874.4)	-71.59 (209.52)	305.23 (95%CI -757.83 to 147.36)

Distribution of NT-proBNP and hs-Crp was skewed and analyses were done by Mann-Whitney two sample test and presented with Hodge-Lehman median (95% confidence interval).

Logarithmic transformed NT-proBNP and hs-Crp and the remaining parameters were compared using an unpaired t-test and presented with the mean difference (95% confidence intervals).

*: Wilcoxon signed rank test P<0.05; #: Two-sample paired t-test P < 0.05; ¤: Two-sample unpaired t-test, P < 0.05. Parameters are presented as mean (SD) and differences in mean (SEM) unless specified otherwise.

### Calcification modulators

#### Fetuin-A

There was a significant period effect on fetuin-A changes (P = 0.01). Therefore, we did not proceed to do any further tests on the effects in the crossover population. The results of each period are presented in Table 2.

**Figure 2:**
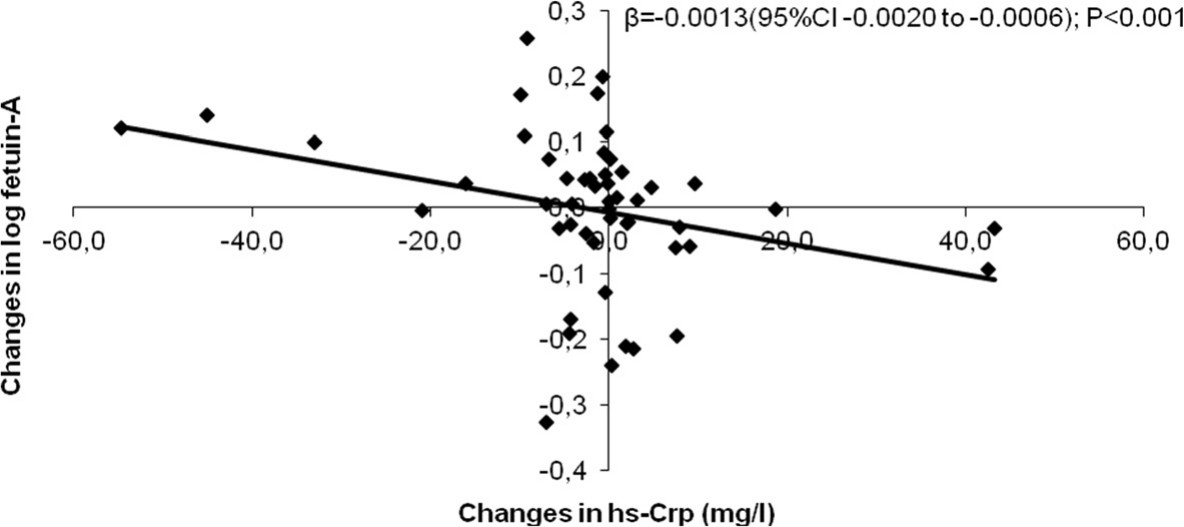
**Changes in log fetuin-A according to changes in hs-Crp.** Univariate analysis of changes in log fetuin-A as an independent variable versus changes in hs-Crp during 16 weeks of treatment with paricalcitol and alfacalcidol.

There was a significant difference in changes in fetuin-A between alfacalcidol and paricalcitol in period 1, owing to a significant increase in fetuin-A in alfacalcidol-treated patients. There were no significant changes within groups or significant differences between groups during period 2.Changes in fetuin-A were logarithmically transformed to satisfy the normality criterion of the parametric tests and analyzed in relation to changes in ionized calcium, phosphate, PTH, FGF23 and hs-Crp using multiple linear regression with backward selection. We found a significant association only between changes in the log of fetuin-A and changes in hs-Crp (Figure 2).

#### OPG

OPG levels in one patient were considered as outliers, as this patient presented extreme measurements that markedly influenced the result. This patient was excluded from further analysis. The patient did not differ from the other patients concerning PTH, ionized calcium or phosphate levels, adverse events or concomitant medication.

No significant period effect or treatment period interaction was found (P = 0.33 and P = 0.18, respectively). Therefore, we proceeded with the analysis for treatment effect. No difference in OPG response between treatment groups was found (P = 0.94).

During period 1, there was a significant increase in OPG in both treatment groups. During period 2 only the alfacalcidol-treated patients had a statistically significant increase in OPG. There was no statistically significant change in OPG during the washout period in either treatment group.

**Figure 3:**
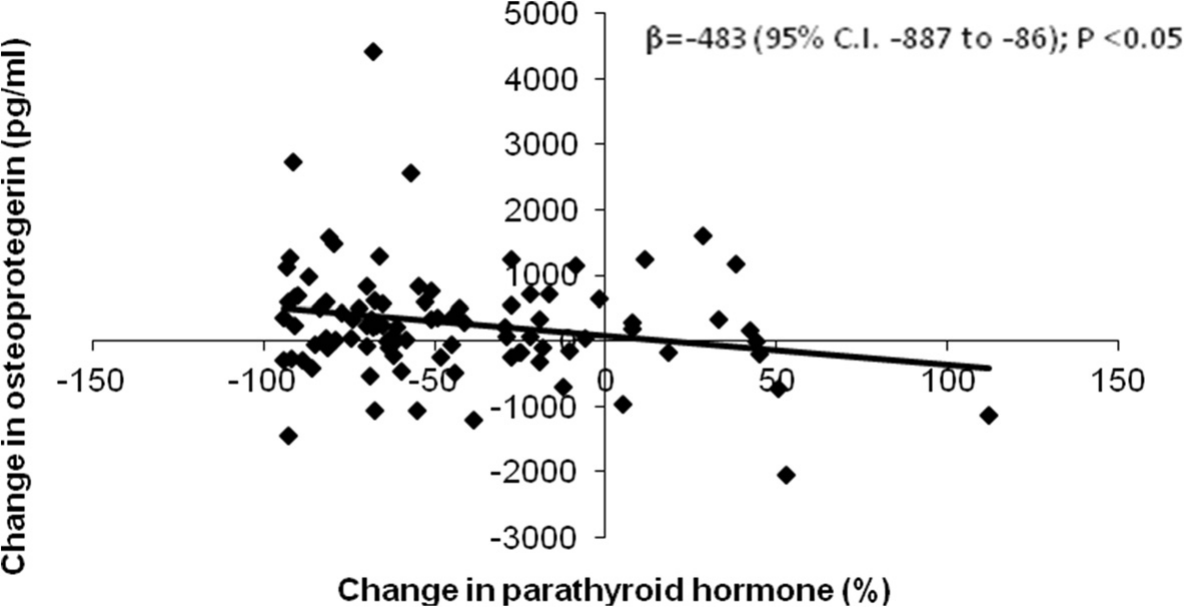
**Changes in osteoprotegerin according to changes in parathyroid hormone.** Univariate analysis of changes in osteoprotegerin as an independent variable versus changes in parathyroid hormone during 16 weeks of treatment with paricalcitol and alfacalcidol.

There was no difference in OPG levels between alfacalcidol and paricalcitol treatment groups at any time (Table 2).Multiple linear regression for repeated measurements was performed to identify factors associated with changes in OPG. Changes in OPG were normally distributed when inspecting histograms and probability plots. No relation between treatment, period, mean final equipotent dose of the vitamin D analog, changes in ionized calcium, phosphate, FGF23 or alkaline phosphatase were found. Equipotent doses of vitamin D analogs were calculated by multiplying the alfacalcidol dose by a factor of 3, because the alfacalcidol:paricalcitol dose-ratio was 1:3. The only factor significantly associated with changes in OPG was the percentage of change in PTH (Figure 3).

#### NT-proBNP

NT-proBNP was logarithmically transformed to satisfy the normality criterion of the parametric tests. No period effect (p = 0.12) or treatment-period interaction (p = 0.07) was found. Therefore, we proceeded to the test of difference between treatment groups and found no significant difference in changes in NT-proBNP between groups (p = 0.15).

During treatment period 1 there was a significant increase in log NT-proBNP in the alfacalcidol-treated group (p < 0.001) and in the paricalcitol-treated group (p < 0.0001).

During treatment period 2 there was a non-significant increase in log NT-proBNP in the alfacalcidol-treated group (p = 0.08) and a non-significant decrease in the paricalcitol-treated group (p = 0.09).

During washout, there were no significant changes in the alfacalcidol-paricalcitol-treated group (p = 0.80), although in paricalcitol-alfacalcidol-treated patients there was a significant decrease in log NT-proBNP (p < 0.05 = 0.0468).

There was no significant difference in changes in NT-proBNP during period 1 in patients with known heart insufficiency at baseline (n = 9) compared with patients with no history of heart insufficiency (n = 46) at baseline (P = 0.22).

There was no correlation between changes in NT-proBNP and changes in FGF23 (P = 0.20) or PTH (P = 0.08).

### Markers of inflammation

#### Hs-Crp

Four patients had hs-Crp levels above 100 mg/l. All of them had a concomitant infection. These four patients (two in the alfacalcidol-paricalcitol group and two in the paricalcitol-alfacalcidol group) were excluded from analysis because the intention was to describe hs-Crp as an inflammatory marker.

The data were logarithmically transformed in order to reach normality. There was no period effect (P = 0.40) or significant treatment-period interaction (P = 0.93). There was no difference between treatment effects (P = 0.76).

#### IL-6

Patients with hs-Crp levels above 100 mg/l who also had high IL-6 levels were deemed outliers and these patients were censored.

There was no period effect (P = 0.85) or significant treatment-period interaction (P = 0.55). There was no difference between treatment effects (P = 0.37).

#### TNF-α

There was no period effect (P = 0.85) or significant treatment-period interactions (P = 0.84) and there was no difference between treatment effects (P = 0.36). The levels of hs-Crp, IL-6 and TNF-α and changes during treatment periods are shown in Table 2.

There were no significant changes in any of the inflammatory markers even after excluding the patients having an infection during the study (n = 11 alfacalcidol-paricalcitol and n = 14 paricalcitol-alfacalcidol).

## Discussion

We found no differences between alfacalcidol and paricalcitol and their influence on NT-proBNP, OPG or inflammatory markers during 16 weeks of treatment in hemodialysis patients. Fetuin-A increased significantly in the alfacalcidol-treated group compared with the paricalcitol-treated group during the first treatment period. Owing to a significant period effect we could not analyze crossover data for differences in changes in fetuin-A.

Fetuin-A was shown in experimental studies to be an inhibitor of vascular calcification [21,22] and low levels of fetuin-A have been associated with increased cardiovascular morbidity and mortality in dialysis patients [23-25]. It is unknown whether an increase in fetuin-A level, as found in the present study, protects against cardiovascular disease.

Fetuin-A is known to be a negative acute phase reactant [26], and in accordance with former cross-sectional studies in dialysis patients demonstrating a negative association between levels of Crp and fetuin-A [25,27], we even found a negative correlation between changes in these parameters. This points to fetuin-A being part of the malnutrition-inflammation-atherosclerosis syndrome in dialysis patients.

We found an increase in fetuin-A during the first treatment period in both groups. This increase was only statistically significantly higher in the alfacalcidol-treated group compared with the paricalcitol-treated group. However, a significant increase in fetuin-A during paricalcitol treatment has been demonstrated after eight weeks of treatment in an uncontrolled study in hemodialysis patients [28]. There was a tendency towards a higher prevalence of patients with diabetes at baseline in the paricalcitol-treated group. This may modulate changes in fetuin-A, as diabetic patients had higher fetuin-A levels and may respond differently than non-diabetic patients [29,30]. The discrepancy between the effects in period 1 and period 2 could be due to a carryover effect from the first treatment period, or the significant difference during period 1 may simply be due to chance. The actual effect of vitamin D analogs on fetuin-A remains to be demonstrated in a placebo-controlled trial.

Alfacalcidol and paricalcitol appeared to increase OPG. This increase may be due to a direct effect of vitamin D analogs or to suppression of parathyroid hormone, which was found to correlate with changes in OPG. Because PTH decreased least in the paricalcitol-treated patients during the second treatment period, this may explain why OPG did not show any significant changes during this period. The changes in OPG during vitamin D analog treatment have, as far as we know, only been studied in an uncontrolled trial of maxacalcitol, where the opposite effects were found, namely that this led to a decrease in OPG [31].

OPG is an important factor in bone metabolism where OPG acts as a decoy receptor for receptor activator of nuclear factor кB (RANK) ligand (RANKL) and inhibits activation of RANK, inhibits maturation of osteoclasts and prevents bone resorption [32]. In animal models, OPG deficiency causes vascular calcification [33] and OPG treatment has been shown to block vascular calcification [34]. However, in dialysis patients, OPG has been associated with calcification and the progression of calcification [35-37]; both in the general population and in the population of chronic kidney disease (CKD) patients, OPG was associated with increased cardiovascular morbidity and mortality [18,38,39]. Whether an increase in OPG during treatment with vitamin D analogs increases the risk of cardiovascular disease or actually reflects a vascular protective mechanism, remains to be further explored.

Left ventricular hypertrophy and cardiac dysfunction are risk factors for cardiovascular mortality in patients with CKD [40]. NT-proBNP predicts cardiovascular and total mortality in hemodialysis patients [41,42]. In experimental models, vitamin D analogs inhibit left ventricular hypertrophy [12,13]. In the PRIMO trial, paricalcitol attenuated the increase in BNP compared with placebo in patients with CKD stages 3–4 [43]. However, in the PRIMO trial and the very similar OPERA trial [44], paricalcitol did not change the left ventricular mass index. In post hoc analysis, a reduction in left atrial volume was detectable after 48 weeks of paricalcitol treatment, which could be an early marker of an increase in left ventricular mass [14]. We explored changes in NT-proBNP during alfacalcidol and paricalcitol treatment in hemodialysis patients and found, as in predialysis patients, a steady increase in NT-proBNP during the intervention period in both treatment groups. The increase in Nt-proBNP may have been attenuated by the vitamin D analogs. Unfortunately, no untreated group was present.

High levels of parathyroid hormone have been associated with left ventricular hypertrophy and high levels of NT-proBNP in patients with CKD and end-stage renal-disease [45]. This may be due to a direct effect of parathyroid hormone on cardiac myocytes [46]. We explored the relation between changes in PTH during treatment with vitamin D analogs and changes in NT-proBNP, and found that the degree of suppression of hyperparathyroidism during 16 weeks of treatment did not influence changes in NT-proBNP.

FGF23 levels are increased as kidney function declines [47] and in dialysis patients treatment with vitamin D analogs increases FGF23 [20]. High FGF23 levels are associated with increased mortality and left ventricular hypertrophy in dialysis patients [48,49]. Experimental studies support that FGF23 induces left ventricular hypertrophy [50]. We found no relation between changes in FGF23 during treatment with vitamin D analogs and changes in NT-proBNP.

The reason why changes in parathyroid hormone or FGF23 do not relate to changes in NT-proBNP may be due to the short intervention period or because these factors are only biomarkers of other mechanisms involved in the pathophysiology of left ventricular hypertrophy and heart failure in patients with CKD.

The malnutrition-inflammation complex are common in dialysis patients and a risk factor for morbidity and mortality [51]. Experimental studies have demonstrated an anti-inflammatory effect of vitamin D [9,52]. We did not find any changes in inflammatory markers during treatment with alfacalcidol or paricalcitol. These findings are in conflict with some previous reports. In a placebo-controlled oral study in CKD stages 1–3 patients [53] and in two uncontrolled (intravenous and oral) studies in dialysis patients, a decrease in hs-Crp, IL-6 and TNF-α was found during paricalcitol treatment [54,55]. However, Moe *et al.* did not find any changes in TNF-α or IL-6 after 12 weeks of treatment with intravenous paricalcitol in a placebo-controlled study in hemodialysis patients with low PTH [56]. Furthermore, 8 weeks of high-dose cholecalciferol did not influence inflammatory markers in predialysis and dialysis patients [57]. Whether an anti-inflammatory effect of vitamin D and its analogs depends on the administration route, dose or patient population including level of hyperparathyroidism, remains to be explored. Furthermore, the present study may be insufficiently powered to detect minor changes in inflammatory parameters.

The present study has several limitations. The study size was small and minor differences may not be detected. Patients participating in the present study were comparable with participants in the main study concerning baseline values; still the missing data may lead to bias, especially in the analysis of uncrossed data. These prevalent patients may differ from incident dialysis patients. Other markers, especially ones involved in calcification, such as matrix-GLA-protein and osteopontin, and direct measurement of vascular calcification using cardiac-CT or abdominal x-ray, may add further information to the influence of vitamin D analogs on calcification in dialysis patients.

## Conclusions

Alfacalcidol may increase the calcification inhibitor fetuin-A compared with paricalcitol in hemodialysis patients. Alfacalcidol and paricalcitol appear to increase OPG to a similar extent during treatment, and NT-proBNP increased equally during both treatments. Both vitamin D analogs did not influence inflammatory markers. Whether these findings are important for patient outcome remains to be further explored.

## Competing interests

DH has received a research grant from Abbott Laboratories for the present study. DH has received lecture fees from Fresenius. LB has received consulting fees from LEO Pharmaceuticals and Amgen and lecture fees from Genzyme, Abbott Laboratories, Leo Pharmaceuticals, Sanofi, Fresenius and Shire.

## Authors’ contributions

DH was the primary investigator of the clinical trial, wrote the study protocol and drafted the manuscript. KR conceived and planned the study, interpreted data and critically revised the manuscript. LMR performed the analysis of OPG, fetuin-A, hs-Crp and NT-proBNP, interpreted data and critically revised the manuscript. HB performed the analysis of IL-6 and TNF-α, interpreted data and revised the manuscript. LB conceived and planned the study, managed the trial, interpreted data and revised the manuscript. All authors read and approved the final manuscript.

## Pre-publication history

The pre-publication history for this paper can be accessed here:

http://www.biomedcentral.com/1471-2369/15/130/prepub
